# Release of growth factors after mechanical and chemical pleurodesis for treatment of malignant pleural effusion: a randomized control study

**DOI:** 10.1515/raon-2015-0002

**Published:** 2015-11-27

**Authors:** Aljaz Hojski, Maja Leitgeb, Anton Crnjac

**Affiliations:** 1 Department of Thoracic Surgery, University Medical Centre Maribor, Slovenia; 2 Biochemistry Division, Medical Faculty, University of Maribor, Maribor Slovenia

**Keywords:** malignant pleural effusion, pleurodesis, growth factors, quality of life

## Abstract

**Background:**

Growth factors are key inducers of fibrosis but can also mediate inflammatory responses resulting in increasing pleural effusion and acute respiratory distress syndrome. The primary aim of the study was to analyse growth factors release after performing chemical and mechanical pleurodesis in the first 48 hours at the patients with malignant pleural effusion. The secondary endpoints were to evaluate the effectiveness of the both pleurodeses, symptoms release and the quality of life of patients after the treatment.

**Patients and methods.:**

A prospective randomized study included 36 consecutive female patients with breast carcinoma and malignant pleural effusion in an intention-to-treat analysis. We treated 18 patients by means of thoracoscopic mechanical pleurodesis and 18 patients by chemical pleurodesis with talcum applied over a chest tube. We gathered the pleural fluid and serum samples in the following 48 hours under a dedicated protocol and tested them for growth factors levels. A quality of life and visual analogue pain score surveys were also performed.

**Results:**

Median measured serum vascular endothelial growth factor (VEGF) level after chemical pleurodesis was 930.68 pg/ml (95% CI: 388.22–4656.65) and after mechanical pleurodesis 808.54 pg/ml. (95% CI: 463.20-1235.13) (p = 0.103). Median pleural levels of transforming growth factor (TGF) β1 were higher after performing mechanical pleurodesis (4814.00 pg/ml [95% CI: 2726.51–7292.94]) when compared to those after performing chemical pleurodesis (1976.50 pg/ml [95% CI: 1659.82–5136.26]) (p = 0.078). We observed similar results for fibroblast growth factor (FGF) β; the serum level was higher after mechanical pleurodesis (30.45 pg/ml [95% CI: 20.40–59.42]), compared to those after chemical pleurodesis (13.39 pg/ml [95% CI: 5.04 – 74.60]) (p = 0.076). Mechanical pleurodesis was equally effective as chemical pleurodesis in terms of hospital stay, pleural effusion re-accumulation, requiring of additional thoracentesis, median overall survival, but, it shortened the mean thoracic drainage duration (p = 0.030) and resulted in a higher symptoms release and in a better quality of life (p = 0.047).

**Conclusions:**

We recorded an increase in serum VEGF levels after chemical pleurodesis, however on the contrary, an increase in the pleural fluid level of TGFβ1 and FGFβ] after mechanical pleurodesis with respect to compared group. Although the differences did not reach statistical significance, VEGF, TGFβ1 and FGFβ remain the most interesting parameters for future research. Considering the mechanisms of growth factors action, we conclude that in our study group mechanical pleurodesis might be more efficient in terms of growth factors release, thoracic drainage duration and resulted in a higher symptoms release and in a better quality of life than chemical pleurodesis.

## Introduction

A large number of different methods for pleurodesis used throughout the world tells us that an ideal procedure is still undetermined. Defining the best palliative treatment for malignant pleural effusion has been elucidated many times.^1^–^3^ In our clinical practice, we use two distinct pleurodesis procedures. The most common choice is chemical pleurodesis with talc. We have also established thoracoscopic mechanical pleurodesis as an alternative method to treat malignant pleural effusions.^4^ By observation, we speculate that chemical pleurodesis causes a systemic inflammatory response whereas mechanical pleurodesis only local tissue response with fewer side effects. Considering the known facts of tissue regeneration, scaring, and healing^5^ we see growth factor release as an important link in these processes.

The primary aim of the study was to analyse growth factors release after performing chemical and mechanical pleurodesis in the first 48 hours. The secondary endpoints were to evaluate the effectiveness of the both pleurodeses, symptoms release and the quality of life of patients after the treatment.

### Growth factors in pleurodesis

Inflammatory cells are the main source of growth factors.^6^ At the same time, we find them attached to the glycoproteins of the extracellular matrix. Different events in the healing process are triggered by their action and interaction.^7^–^9^ Their role in the process of pleurodesis was studied many times.^10^–^12^ A research for their differential diagnostic power has exposed the vascular endothelial growth factor (VEGF) being typically higher in malignant pleural effusion.^13^ We must also take into account and explore the impact of their systemic effects, particularly the role of VEGF in relation to the acute respiratory distress syndrome.^14^,^15^

VEGF induces angiogenesis as well as increases vascular permeability and stimulates the tumour growth and metastasis.^16^ It promotes the formation of pleural effusion and is a crucial factor for the growth of malignant tissue and formation of metastases.^17^–^19^ A study on an animal model showed the transforming growth factor (TGF) β1 to be an ideal inductor of pleurodesis with a better performance than talc but minimal to no side effects.^12^ Fibroblast growth factor (FGF) β is clearly linked to the success of pleurodesis.^10^ After damaging the integrity of extracellular matrix, it is released from the binding sites and serves as the first inductor of fibroblast proliferation and collagen synthesis.

## Patients and methods

The study included 36 female patients with breast carcinoma and cytologically confirmed malignant pleural effusion whose lungs re-expanded after thoracic drainage and were eligible for surgery. The patients had the Eastern Cooperative Oncology Group (ECOG) performance status 0–2. The study was approved by the National Ethics Committee with number 40/09/09 and conducted at the Department of Thoracic Surgery at the University Medical Centre Maribor, Slovenia, between July 2010 and August 2013. All patients signed a written consent to participate in the study. Laboratory tests and statistical analysis were carried out at the same centre in cooperation with the Biochemistry Division of Medical Faculty, University of Maribor.

Overall, 81 female patients with breast carcinoma and malignant effusion were presented by the oncologists, of whom 36 met the inclusion criteria. We excluded from the study patients who were due to an underlying disease or concomitant diseases not fit to undergo surgery under general anaesthesia or had a trapped lung. Their demographic data and history as well as prognostic factors that could affect the treatment outcome in different oncological patients (age^20^,^21^, type of tumour^16^,^22^, time interval from previous surgical treatment till pleurodesis, specific systemic oncological therapy^22^,^23^, performance status^22^,^24^, maximal volume of previous thoracentesis^25^) were collected (Table 1).

A random numbers were assigned to the patients at admission and they were divided into two groups. Group with chemical pleurodesis with talc (n = 18) and group with thoracoscopic mechanical pleurodesis (n = 18).

Patients with chemical preurodesis were treated with a 5 g of talcum (Ph.Eur.7.0, Caesar Loretz GmbH, Austria, EU) and 100 ml of 0.9% NaCl slurry over the chest drain. We administered 40 ml of 1% lidocaine intrapleuraly, 20 to 30 minutes prior to talk application. Next, we clamped the drain for 2 hours and then reattached it to the active suction (−15 cm H_2_O) drainage system for 24 hours. After the first day, the drainage system was switched to underwater seal gravity drainage until the daily amount of drained fluid was less than 200 ml. A favourable chest x-ray report was a requirement for chest tube removal.

Patients with mechanical pleurodesis were treated with a two-port video-assisted thoracoscopic surgery (VATS)^26^ during which we performed the mechanical abrasion of parietal pleura using A.M.I.-dock reusable applicator for disposable (DLU) tips (A.M.I. GmbH, Austria, EU).

During VATS, we assessed the coverage of the pleura with malignant tissue:

0 = no obvious lesions;
1 = isolated lesions;
2 = diffuse (covering the majority of the pleura) lesions;
3 = massive (normal pleura could not be seen).

The result was calculated by adding the data from visceral, parietal and diaphragmal pleura and ranged from 0 to 9 (Table 1).

After 24 hours of active suction (−15 cm H_2_O) drainage, we disconnected the drains to allow underwater seal gravity drainage of effusion and removed them after a favourable chest x-ray when the daily amount of drained fluid was less than 200 ml.

Post procedural analgesia was kept the same for both study arms. We used a combination of parenteral metamizole, piritramide and after the first post procedural day, we switched to per orally administered combination of paracetamol and tramadol.

To assess the growth factors release we used a specially designed protocol based on research data of our own unpublished pilot study. The time frame for sampling the pleural fluid and blood samples was: prior to the procedure (time 0) and 3, 12, 24, 36 and 48 h after procedure. For both groups we used only one 20F silicone chest drainage catheter (Portex, Smiths Medical, USA) oriented posteriorly and caudally, through which we collected pleural fluid samples. Blood samples taken at 24 and at 48 hours were tested for two general indicators of inflammation: leukocyte count, C-reactive protein (CRP) level. All pleural samples were also tested for lactate dehydrogenase (LDH) level and pH level of the effusion was determined before the pleurodesis. The supernatant prepared from the pleural fluid samples was centrifuged for 10 min at 3000 revolutions per minute (RPM) at 4°C. Alongside with that of the blood serum it was frozen at −70°C and stored for later ELISA testing to determine FGFα and β, VEGF and TGFβ1 levels.

To evaluate the effectiveness of both methods of pleurodesis we used chest X-ray and pleural cavity ultrasound examination. The length of thoracic drainage, hospital stay and pleural effusion re-accumulation were recorded (Table 2). A need for additional procedures due to the recurrence of dispnoic problems and pleural effusion accounted for an unsuccessful pleurodesis. The overall survival was calculated from the day of pleurodesis till the death for any causes. Follow-up lasted up to 12 months.

To describe better the immediate effects of pleurodesis, we measured the visual analogue pain score (VAS) at 0, 12, 24, and 48 hours after pleurodesis. Additionally, we used the questionnaire of European Organisation for Research and Treatment of Cancer (EORTC) QLQ-C30, version 3.0^27^ to determine the impact of treatment on patients’ quality of life. Global health status, physical functioning, fatigue, pain and dyspnoea measurements were selected. Patients completed them with the data for the week before the procedure and for the week and month thereafter. These questionnaires served us as a reflection of patient tolerance towards pleurodesis.

### Statistical analysis

Statistical analysis was performed by using IBM SPSS Statistics for Windows, Version 19.0. Armonk, NY: IBM Corp. When comparing groups of patients, χ^2^ test was used to test the frequency counts, t-test was used to test the means of continuous variables and when the value distribution was not normal, Mann-Whitney U test was used to test their median values. Kolmogorov-Smirnov test was used for assessing the normality of data. When dealing with repeated measures, mixed ANOVA was used to compare the mean values between and within groups. Kaplan-Meier survival analysis was used for estimating the survival function for both groups. When performing multiple comparisons Bonferroni correction was used to adjust p-values. Statistical significance was set at p < 0.05.

## Results

### Patients

The two observed groups did not differ significantly with respect to demographic data and prognostic factors (Table 1). The age, hormonal status of tumours, HER2 status, and time interval from previous surgical treatment, last chemotherapy, time from diagnosis of malignant pleural effusion until pleurodesis, specific systemic oncological therapy, performance status and maximal volume of previous thoracentesis were well balanced (Table 1).

The average coverage of the pleura in the group with mechanical pleurodesis was 3.4 out of 9 points and the median value was 3. The pH values of pleural effusions did not statistically differ (Table 1).

### Growth factors release after performing chemical and mechanical pleurodesis

**FIGURE 1.**
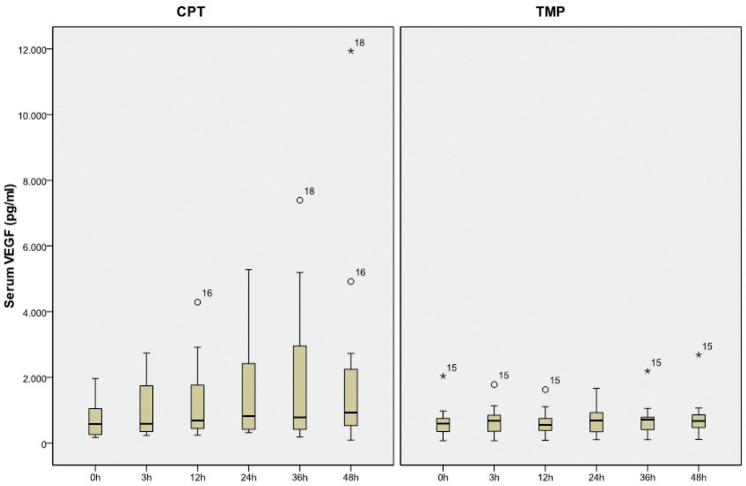
Comparison of serum VEGF values during 48 hours after chemical (CPT) and mechanical (TMP) pleurodesis (p = 0.103).

The focal point of our study was serum VEGF, specifically the maximum value for a given patient. Median serum VEGF level, measured after chemical pleurodesis, was 930.68 pg/ml and after mechanical pleurodesis, it was 808.54 pg/ml (Table 2, Figure 1). Maximal serum VEGF level after chemical pleurodesis was 11930 pg/ml and after mechanical it was 2687 pg/ml (Figure 1).

**FIGURE 2.**
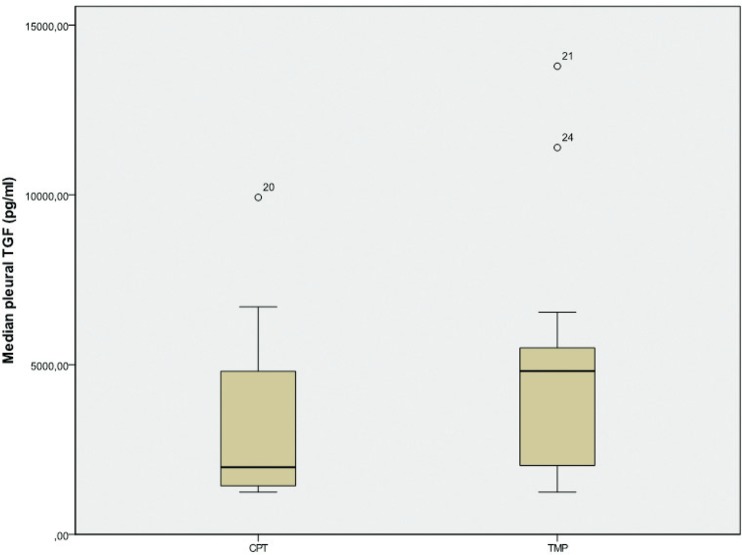
Comparison of median values of pleural TGF β1 during 48 hours after chemical (CPT)-1 and after mechanical (TMP) pleurodesis – 2 (p = 0.078).

Other key values that showed intergroup difference were that of the pleural TGFβ1 and FGFα and β. Median pleural levels of FGFα and β and TGFβ1 were higher after performing mechanical pleurodesis, when compared to those after performing chemical pleurodesis (Table 2, Figure 2).

Levels of VEGF in pleural fluid samples and TGFβ1 in serum were similar in both groups.

We have not recorded any specific impact of any technique of pleurodesis on the serum levels of FGFα and β, most of the time they were undetectable being below the lower limit of sensitivity of ELISA.

We found the average largest increase in serum VEGF, TGFβ1 pleural value, FGFα and β pleural values between the 24^th^ and 36^th^ hour post pleurodesis. VEGF levels in the serum were still growing up to 48^th^ hour post pleurodesis when we stopped taking samples.

There was a significant elevation of blood leukocytes in the first 24 hours, it was more pronounced for the group with chemical pleurodesis. The same was established for the CRP levels. The LDH levels in serum and pleura correlated well with the levels of inflammation and they show a tendency to be lower after mechanical pleurodesis compared to those after chemical pleurodesis (Table 3).

We made no relevant correlation between pH value of pleural effusion and success of pleurodesis or level of growth factors.

### The effectiveness of pleurodesis

Both methods were successful in preventing the recurrence of malignant pleural effusion. One patient in the group with mechanical pleurodesis and two patients in the group with chemical pleurodesis needed additional interventions due to the re-accumulation of MPE. The difference in the pleurodesis success between the groups was not significant as in other previous studies on small samples, except in terms of length of post procedural thoracic drainage (Table 4). Mean thoracic drainage duration after mechanical pleurodesis was significantly shorter than after chemical pleurodesis (p = 0.030).

We found no relevant correlation between pleurodesis success and the level of growth factors in our study groups.

One patient in the group with mechanical pleurodesis had a larger drainage of bloody effusion. Haemoglobin in the pleural fluid sample was 4.6 g/dL - 800 ml on the first postoperative day. The volume of drainage has significantly decreased on the post-operative day one. One patient from the group with chemical pleurodesis formed an empyema by the end of the first week after pleurodesis. We treated her successfully using negative-pressure wound therapy (V.A.C. freedom, K.C.I., USA) over the thoracostomy at the site of the chest drain for 25 days.

The median survival of patients was 6.8 months after performing chemical and 7.0 months after performing mechanical pleurodesis (p = 0.060) These patients were evenly distributed between both observation groups and Kaplan-Meier survival analysis showed similar survival curves for both groups. Five patients survived for 1 year or more, which shows more than 10% of one-year survival. There was also no mortality associated with the reported procedures (Table 4).

### Symptoms release and the quality of life

The measured score through a pain visual analogue scale (VAS) showed lower pain-load for patients in the TMP group (Table 5). There was significant less pain load after mechanical pleurodesis compared to chemical pleurodesis 12 hours post procedure (p = 0.039). This difference was lost after 48 hours.

We got similar results for quality of life (Table 6). The quality of life questioners were filed at the three time points: (1) “pre” – one week before pleuodesis, (2) “post” - 1 week after pleurodesis, and (3) “end” – 1 month after pleurodesis. Tables 6 and 7 summarize the important results to express how the observed interventions effect patients and the impact of the treatment on the quality of life when the effect of pleurodesis is maximal. For specific indicators we took global health, physical functioning, fatigue, pain, and dyspnoea.

In both groups, we saw an improvement in quality of life over time. For the majority of targeted questions, the comparison between the two groups showed a statistically significant improvement of quality of life in the group with mechanical pleurodesis. A distinctly beneficial effect of mechanical pleurodesis was present as a reduction in the form of dyspnoea (Table 7).

## Discussion

It is our strong belief that studies oriented towards pleural space can help us understand many problems we encounter in our daily work. An ideal agent that would produce effective pleurodesis in the shortest possible time with little side effects and would be affordable remains vague. Light wrote in an article summarizing his research on pleural space that it is the lack of interest of industry that turns researchers away from dedicated research.^28^

Our wish was to set up new, additional standards on how to determine the best agent for this palliative procedure. We are focused on the culprits for small but significant differences between two methods of pleurodesis and try to explain the reasons for rare but serious side effects after chemical pleurodesis with talcum. For patients with malignant pleural effusion, a pleurodesis efficiency of more than 90% achieved by the chemical pleurodesis is satisfactory and does not necessarily require further studies. However, the process of healing and scarring is extremely important for understanding the impact of surgical procedure on the body and opens up many opportunities for improvement. Progress in methods of pleurodesis can be used as an advantage for patients with recurrent primary pneumothorax and other cases where chemical pleurodesis is often used although many thoracic surgeons believe that this is not the best choice of treatment.

Besides the usual indicators of inflammation, such as leukocyte number, LDH or CRP^14^, we are establishing growth factors as additional parameters to determine the safety and efficiency of different methods of pleurodesis.

VEGF is the most extensively studied cytokine related to pleural effusion and pleurodesis outcome; it has numerous actions crucial to our understanding of the effects of pleurodesis, central being the fact that it is 50,000 times more potent vasodilator than histamine.^29^ Previous research data show that an ideal pleurodesis should have limited excretion of VEGF into the pleural space, but should not elevate its serum levels.^15^,^17^–^19^,^28^,^29^ VEGF is related to the increased permeability of pleura and promotes growth and metastasis of malignancies.^15^,^16^,^18^,^19^ We believe that accelerated release of VEGF after chemical pleurodesis with talc gives serious concerns for its use.^18^,^29^ This is especially important for patients with increased risk of respiratory problems and maybe generally for patients with malignancies. Extremely high serum levels of VEGF were observed in a small proportion (10%) of patients following chemical pleurodesis. These values of serum VEGF significantly exceed those for which it has been shown that are dangerously high.^15^

TGFβ1 is another multifunctional cytokine strongly connected to success of pleurodesis; in areas of inflammation, it stimulates cell proliferation, increases mesothelial permeability, and the production of fibrin, collagen, and tissue fibrosis.^12^ Experimental studies have also shown that corticosteroids and nonsteroidal anti-inflammatory drugs (NSAIDs) reduce the extent of talcum or doxycycline pleurodesis, but, these drugs have no influence on pleurodesis induced with TGF β1.^30^ FGFβ is another potent inductor of angiogenesis and fibrosis we see relevant.

Female patients with breast cancer and associated malignant pleural effusions are the most appropriate population to answer our research question. Their survival is relatively good and the patients are at the presentation of pleural effusions in a good performance status.^31^ A large caseload enables us to strictly adhere to the main inclusion criteria: good performance status, positive cytology and re-expanded lung after evacuation of pleural effusion. The need for many repeated thoracenteses, regardless of positive cytology in a patient with breast cancer, is usually an indication for chemical pleurodesis. However, mechanical pleurodesis is another possible method for the prevention of malignant pleural effusion recurrence, but, not widely used. In our study, the two methods of pleurodesis show distinctly different features. Mechanical pleurodesis has on the contrary to chemical considerably less side effects and is proven more effective in advanced malignancy.^4^

Our data was collected on a small sample, but, we can plan for broader studies with targeted sample collection between 24 and 48, or up to 72 hours after the procedure. Monitoring and comparison of specific biochemical parameters is reasonable on a small number of well-comparable patients as well^29^, which we guaranteed with the specified inclusion criteria. The timelines were set based on previous studies^4^ and a pilot study that we conducted at our department prior to the study that is presented. A measurable increase of growth factors levels in blood serum and pleural fluid is unlike in animal studies^14^ observed a little later. We recorded the largest increase of serum VEGF and pleural TGFβ1 and FGFα and β values between the 24^th^ and 36^th^ hour. After this period, the values were still gradually increasing up to 48 hours post pleurodesis when we stopped recording them. These processes might be lengthier in humans compared to small animals, which should be considered for future research. The collection of samples after 48 hours is difficult as pleurodesis is usually already quite strong and the amount of fluid drained through catheters, that may already be clogged, is minimal.

There was also no relevant correlation between success of pleurodesis and the level of growth factors in our study groups. We believe this is due to the high efficiency of both methods. Therefore, it is difficult to estimate the impact growth factors have in these few unsuccessful cases. We made our conclusions based on our study in the light of several other reports that describe the role of grow factors in the setting of pleural effusion and pleurodesis.

The two observed methods, chemical and mechanical pleurodesis, are the most commonly used for treatment of patients with primary spontaneous pneumothorax. Small amounts of drained pleural effusion after pleurodesis in this group prevent an insight into the release of cytokines and other components that contribute to fibrosis. In addition, we chose patients with malignant pleural effusion as a more appropriate observation group as we do not perform chemical pleurodesis on young patients or for benign diseases with long-term survival, such being the primary spontaneous pneumothorax.

The coverage of the pleura with malignant tissue is another factor that affects the success of pleurodesis, which we could verify only in the group with mechanical pleurodesis. The fact that the lungs re-expanded, normal width of the mediastinum on the chest x-ray and positive cytology allows us to conclude that in the patients group with chemical pleurodesis the pleura was also covered with a comparable proportion of malignant tissue. A small number of unsuccessful pleurodesis in our study is attributed to the selection criteria, since the fibrosis process is more pronounced in healthier pleura. Judging by the data collected in the group with mechanical pleurodesis, an average coverage is only 3.4 from a total of 9 points.

Our protocol included questionnaires on pain load and quality of life. The data are relevant, so we included them in this paper. Patients reported greater sensation of pain and dyspnoea after performing chemical compared to the mechanical pleurodesis. We see pleural inflammation as the main source of pain after pleurodesis. Additionally, we are connecting the effect, which talcum has on the lung parenchyma, with the sensation of dyspnoea. After being absorbed to some extent into tissue, talc causes pneumonitis, pulmonary oedema and signs of acute respiratory distress syndrome. This effect of pleurodesis is less pronounced after mechanical pleurodesis.

There was a significant main effect of time on all assessed quality of life scales (global health status, physical functioning, fatigue, pain and dyspnoea) with “post” scores being significantly better than “pre” scores and “end” scores being better than “post” scores with only one exception, there was no significant difference between “pre” and “post” dyspnoea score. There was also a significant main effect of treatment group on the global health status, fatigue and pain score with better average scores across all points in time in the TMP group. We were especially interested in the interaction effect between time and treatment group, broken down to comparing “post” and “end” scores to “pre” scores across both groups. These comparisons revealed significant interactions when comparing chemical and mechanical pleurodesis global health status scores (both “post” vs. “pre” and “end” vs. “pre”), “end” vs. “pre” fatigue scores and “end” vs. “pre” dyspnoea scores. Looking at the interaction graphs, this suggests that those scores improved more substantially from “pre” to “end” point in the group with mechanical pleurodesis than in the group with chemical pleurodesis as well as from “pre” to “post” point in the case of global health status (Tables 6, 7).

By testing our ideas and views of treatment of malignant pleural effusion, we tried to look into the effect our treatment has on tissue or on the whole body. By giving some answers as to what is important in view of better and faster healing and what should be omitted when testing new methods, we try to help the dedicated laboratories. These parameters can be easily recorded in laboratory animals and can help in minimising the needed numbers. We are also directing the interest of professional public towards the growth factors when trying to explain other differences between surgical treatments.^31^ New solutions for many clinical issues can be developed by examining the process of healing and fibrosis.

We believe that the main limiting factor for the clinical use of growth factors is the cost. It is especially limited for terminal patients. Nevertheless, for further experimental studies on pleurodesis and other procedures that boost or stimulate fibrosis growth factors represent an important indicator of efficiency and safety. It is also interesting that TGF β1 works better in an environment with a lower pH.^32^ Again this experimental data could not be clarified in our study groups, mainly because the success rate was very high. It would probably be necessary to perform autopsies to reveal the extent of fibrosis and connect this data to the level of pH and the TGF β1 level. In cases of malignant pleural effusion, a low pH value is an indicator of an advanced disease, such patients accumulate effusion faster. In this case, an agent that triggers abundant secretion of TGF β1 should enable faster pleurodesis.

Surgeons use extremely expensive devices and procedures to promote healing and fibrosis. We believe that further exploration of clinical application or promotion of topical secretion of growth factors might be useful in many cases. One option is by supplying the growth factors to the place where we want their activities. Another would be the promotion of targeted growth factors secretion that could accelerate healing. Both should be useful on the resection surfaces and reduce the time of tissue leakage and bleeding. Achieving this might have a significant impact on the main factors for the postoperative complications and duration of hospitalization.

## Conclusions

We record ed an increase in serum VEGF levels after chemical pleurodesis with talcum and an increase in the pleural fluid level of FGFβ and TGFβ1 after thoracoscopic mechanical pleurodesis with respect to compared group. The differences did not reach statistical significance; however, TGFβ1, FGFβ and VEGF remain the most interesting parameters for future research. Considering the mechanisms of growth factors action, we conclude that in our study group mechanical pleurodesis might be more efficient in terms of growth factors release, better-tolerated and safer method than chemical pleurodesis.

## Figures and Tables

**TABLE 1.** Demographical and specific data related to preoperative conditions for chemical pleurodesis with talc (CPT) and thoracoscopic mechanical pleurodesis (TMP) group group

	**CPT (n = 18)**	**TMP (n = 18)**	**p-value**
Age (years) [±SD]	66 [± 12]	63 [±11]	0.418
Positive hormone receptors (estrogen/progesterone)	55.6%	50.0%	0.757
HER2-positive	31.4%	23.9%	0.741
Time interval from surgery till pleurodesis (months) [±SD]	63 [± 45]	66 [± 53]	0.884
Time interval from chemotherapy till pleurodesis (months) [±SD]	6.5 [± 4.0]	7.0 [± 5.0]	0.837
Received chemotherapy before pleurodesis	94.4%	95.0%	1.000
Received radiotherapy before pleurodesis	33.3%	55.5%	0.210
Median ECOG performance status	1	1.5	0.635
Time from diagnosis of malignant pleural effusion to pleurodesis (months) [±SD]	3 [± 1]	5 [± 2]	0.112
Max. volume of previous thoracocentesis (ml) [±SD]	1297 [±354)	1375 [±441]	0.555
Pleural dissemination (median value)	not measurable	3	-
pH [±SD]	7.27 [±0.09]	7.22 [±0.06]	0.072

ECOG = Eastern Cooperative Oncology Group; HER2 = human epidermal growth factor receptor 2; SD = standard deviation

**TABLE 2.** Median values of growth factors in serum and pleural fluid after chemical pleurodesis with talc (CPT) and thoracoscopic mechanical pleurodesis (TMP)

	**CPT (n = 18)**	**TMP (n = 18)**	**p-value**
Maximum value of serum VEGF (pg/ml) [95% CI]	930.68 [388.22 - 4656.65]	808.54[463.20 – 1235.13]	0.103
Median value of pleural TGFβ1 (pg/ml) [95% CI]	1976.50 [1659.82 – 5136.26]	4814.00 [2726.51 – 7292.94]	0.078
Median value of pleural FGFα (pg/ml) [95% CI]	108.35 [73.29 – 162.62]	66.265 [60.71 – 153.12]	0.364
Median value of pleural FGFβ (pg/ml) [95% CI]	13.39 [5.04 – 74.60]	30.45 [20.40 – 59.42]	0.076

CI = confidence interval; FGF = fibroblast growth factor; TGF = transforming growth factor; VEGF = vascular endothelial growth

**TABLE 3.** Serum inflammatory parameters and biochemical parameters of pleural effusion before and after chemical pleurodesis with talc (CPT) and thoracoscopic mechanical pleurodesis (TMP)

	**CPT (n=18)**	**TMP (n=18)**	**p-value**
Mean increase in serum leucocyte value (x10^9^/l) [± SD] (median value of increase)	3.8 [±3,3] (3.0)	2.9 [±3.1] (2.3)	0.456
Mean increase in serum CRP value (mg/l) [± SD] (median value of increase)	54 [±46] (36.5)	61 [±59] (34.5)	0.977
Mean increase in pleural LDH value (μkat/l) [± SD] (median value of increase)	5.3 [±7.6] (2.8)	4.6 [±18.5] (3.5)	0.826

CRP = C-reactive protein; LDH = lactate dehydrogenase; SD = standard deviation

**TABLE 4.** Results of post pleurodesis (PD) outcomes for chemical pleurodesis with talc (CPT) and thoracoscopic mechanical pleurodesis (TMP) group

	**CPT (n = 18)**	**TMP (n = 18)**	**p-value**
Mean thoracic drainage duration (days)	4.5	3.8	**0.030**
Median hospital stay (days)	5	5	0.126
Pleural effusion re-accumulation	38.9%	20.0%	0.288
Additional thoracentesis required	11.1%	5.0%	0.595
Median survival post PD (months) [95% CI]	6.8 [5–9]	7.0 [4–10]	0.060

CI = confidence interval

**TABLE 5.** Median visual analogue scale (VAS) score for pain before and after chemical pleurodesis with talc (CPT) and thoracoscopic mechanical pleurodesis (TMP)

	**CPT (n=18)**	**TMP (n=18)**	**p-value**
Median VAS before pleurodesis [95% CI]	2 [2–3]	3 [2–3]	0.311
Median VAS 12 hour [95% CI]	5 [3–6]	4 [2–4]	**0.039**
Median VAS 24 hour [95% CI]	5 [4–6]	4 [3–5]	0.085
Median VAS 48 hour [95% CI]	3 [3–5]	3,5 [3–4]	0.881

CI = confidence interval

**TABLE 6.** Comparison of quality of life questionnaire results for global health, physical functioning, fatigue, pain, and dyspnoea between groups at 3 different points in time according to the pleurodesis (PD) - chemical pleurodesis with talc (CPT) and thoracoscopic mechanical pleurodesis (TMP)

	**CPT (n = 18)**			**TMP (n = 18)**		
	
	**pre PD**	**post PD**	**end PD**	**pre PD**	**post PD**	**end PD**
Global health status (mean ± SD/median)	15.7 ± 9.0/16.7	20.4 ± 10.0/16.7	28.7± 14.1/25.0	20.0 ± 11.6/16.7	31.3 ± 7.1/33.3	43.8± 12.9/41.7
Physical functioning (mean ± SD/median)	38.9 ± 11.5/40.0	47.0 ± 20.6/46.7	48.9 ± 21.1/53.3	45.0 ± 14.8/40.0	51.3 ± 12.3/53.3	64.0 ± 13.4/66.7
Fatigue (mean ± SD/median)	77.2 ± 15.9/77.8	68.5 ± 18.4/66.7	64.2 ± 20.4/66.7	70.6 ± 17.0/77.8	56.7 ± 16.5/55.6	41.1 ± 19.8/38.9
Pain (mean ± SD/median)	67.6 ± 20.2/66.7	63.0 ± 14.6/66.7	52.8 ± 21.6/58.3	64.2 ± 16.5/66.7	55.8 ± 13.5/50.0	35.0± 17.0/33.3
Dyspnoea (mean ± SD/median)	68.5 ± 21.3/66.7	61.1 ± 26.2/66.7	63.0 ± 25.3/66.7	76.7 ± 21.9/66.7	65.0 ± 20.2/66.7	40.0 ± 20.5/33.3

pre PD = 1 week prior to pleurodesis; post PD = 1 week after the pleurodesis; end PD = 1 month after pleurodesis

**TABLE 7.** Comparison of the quality of life questionnaire results between the chemical pleurodesis with talc and thoracoscopic mechanical pleurodesis group

	**Main effect of time p - value**	**Main effect of treatment p - value**	**Interaction effect between time and treatment (pre to post treatment) p - value**	**Interaction effect between time and treatment (pre to end result) p - value**
Global health status^*^	< 0.001	0.001	**0.032**	**0.047**
Physical functioning	< 0.001	0.057	0.742	0.083
Fatigue	< 0.001	0.008	0.271	**0.014**
Pain	< 0.001	0.040	0.477	0.057
Dyspnoea	< 0.001	0.536	0.631	**< 0.001**

* Mauchly’s test indicated that the assumption of sphericity had been violated for global health status; therefore, Greenhouse-Geiser correction was used
